# Isolation and Proteomics of the Insulin Secretory Granule

**DOI:** 10.3390/metabo11050288

**Published:** 2021-04-30

**Authors:** Nicholas Norris, Belinda Yau, Melkam Alamerew Kebede

**Affiliations:** Charles Perkins Centre, School of Medical Sciences, University of Sydney, Camperdown, NSW 2006, Australia; nicholas.norris@sydney.edu.au (N.N.); melkam.kebede@sydney.edu.au (M.A.K.)

**Keywords:** insulin secretory granule, beta-cells, granule protein purification

## Abstract

Insulin, a vital hormone for glucose homeostasis is produced by pancreatic beta-cells and when secreted, stimulates the uptake and storage of glucose from the blood. In the pancreas, insulin is stored in vesicles termed insulin secretory granules (ISGs). In Type 2 diabetes (T2D), defects in insulin action results in peripheral insulin resistance and beta-cell compensation, ultimately leading to dysfunctional ISG production and secretion. ISGs are functionally dynamic and many proteins present either on the membrane or in the lumen of the ISG may modulate and affect different stages of ISG trafficking and secretion. Previously, studies have identified few ISG proteins and more recently, proteomics analyses of purified ISGs have uncovered potential novel ISG proteins. This review summarizes the proteins identified in the current ISG proteomes from rat insulinoma INS-1 and INS-1E cell lines. Here, we also discuss techniques of ISG isolation and purification, its challenges and potential future directions.

## 1. Insulin Granule Biogenesis and Function

The insulin secretory granule (ISG) is the storage vesicle for insulin in pancreatic beta-cells. It was long treated as an inert carrier for insulin but is now appreciated as a regulatory structure all on its own. There is a continuous turnover of insulin granules in the beta-cell, which is highly specialised in its capacity for ISG biogenesis, and insulin represents the most abundant protein within the beta-cell at 5–10% of total cell protein mass [1]. Production of insulin first begins in the rough endoplasmic reticulum with the synthesis of preproinsulin [2]. The signal peptide of preproinsulin is cleaved to form proinsulin, which is folded and trafficked to the Golgi complex [3]. Here, proinsulin is packaged with other proteins destined for secretion into a budding immature ISG at the *trans*-Golgi network via a mechanism termed ‘*sorting by entry*’ [4]. Following the release of these granules from the *trans*-Golgi network, maturation of the immature ISG includes acidification of the granule lumen by ATP-dependent proton pumps and promotion of endoprotease convertases (PC1/3 and PC2) activity that cleave proinsulin to form free C-peptide and mature insulin, comprised of the A and B chains bound together by two inter-chain disulfide bonds [5,6,7]. Through a secondary mechanism called ‘*sorting by retention*’, proinsulin and other proteins are retained in the immature ISG (Davidson et al., 1988), while in parallel, proteins such as clathrin are removed from the immature ISG via ‘*sorting by exit*’ [8,9]. Finally, insulin crystallises with zinc cations (Zn^2+^), assembling an ~300 nm dense-core mature ISG [10]. From its point of synthesis, proinsulin enters an ISG within 4 hours [5] and is processed into insulin in a mature ISG within 40 minutes [9].

**Figure 1:**
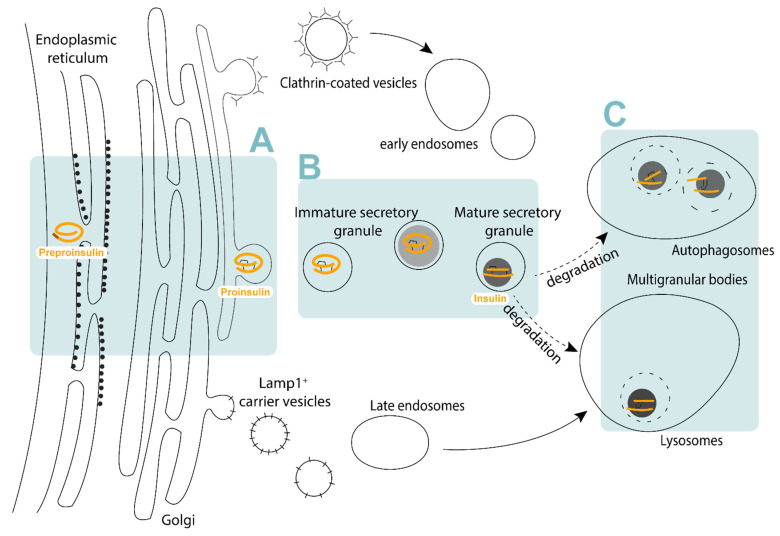
The insulin secretory pathway. (**A**) Proinsulin is synthesised and folded at the endoplasmic reticulum, trafficked to the trans-Golgi network and sorted into budding immature insulin secretory granules. (**B**) Immature insulin secretory granules undergo maturation where proinsulin is cleaved into mature insulin and condenses with zinc to form the dense core within the mature insulin secretory granule. (**C**) Aged mature insulin secretory granules that do not undergo secretion are trafficked for degradation within lysosomes or autophagosomes.

The ISG has a half-life of 3–5 days within the beta-cell cytoplasm [11] (Figure 1), and are ultimately destined for secretion or degradation. Upon glucose stimulation, ISG are motivated to undergo exocytosis, which requires the coordination of cellular machinery present both on ISGs and at the plasma membrane. It is therefore likely that ISG composition contributes to exocytosis, though the variables that determine whether an ISG eventually undergoes secretion are still unclear. Only 1–2% of total ISG content is released upon a single glucose stimulation [12]. Plasma membrane proximity [12,13] and docking [14,15] have long been suggested to contribute to an ISG’s secretory capacity. More recently, ISG motility [16] and age [16,17,18] have also been shown to significantly contribute to an ISG’s propensity for translocation to the plasma membrane and its necessity for docking [16,18]. Finally, an ISG’s fusion capacity–whether the ISG collapses or is recycled–may also be intrinsically regulated [19]. 

ISGs that are not secreted are targeted to the lysosome for degradation, either through autophagosome-dependent or independent pathways [20]. As insulin accounts for a large proportion of protein synthesis in pancreatic beta-cells [21], ISG homeostasis is essential to maintaining beta-cell function [22]. In autophagosome-dependent degradation, ISGs are engulfed by autophagosomes and subsequently fuse with the lysosomes, degrading ISG contents [22,23]. Autophagosome-independent degradation involves the fusion of ISGs with the lysosomes directly (crinophagy) [24]. Apart from whole ISG degradation, many proteases involved may also directly influence insulin turnover. For example, insulin has been shown to be degraded by insulin-degrading enzyme (IDE) in beta-cells and deletion or inhibition of this enzyme perturbs insulin secretion in beta-cells [25,26].

It is now appreciated that all these processes are not only externally regulated by the ISG environment, and proteins both in and on ISG can modulate both the processing and trafficking of ISGs, ultimately controlling granule mobility, secretion capacity, and degradation. Our current review focuses on the continuing pursuit to characterise ISG-localised proteins from pancreatic beta-cells.

**Figure 2:**
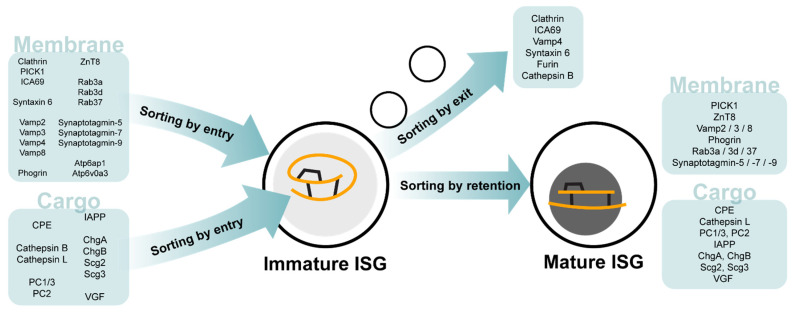
ISG membrane and cargo proteins of the immature and mature ISG. ISG proteins associated with the *‘sorting by entry*’, ‘*sorting by exit*’ and ‘*sorting by retention*’ steps of ISG biogenesis and maturation.

The ISG is key to beta-cell identity. Pathological dysfunction related to insulin occurs at all stages, from synthesis to secretion and primarily results in diabetes. Loss of ISG in beta-cells, termed degranulation, is particularly characteristic of Type 2 diabetes (T2D) and recognised as a marker of beta-cell failure. It is most commonly visualised as a loss of insulin content [27] and seen as a precursor to beta-cell dedifferentiation [28]. Degranulation may occur at the point of SG biogenesis, such as in instances of chromogranin B (CgB) deficiency [29] or the loss of vacuolar sorting protein 41 [30], which regulate ISG budding and ISG coat formation respectively. Alternatively, degranulation may also be the result of chronic overnutrition leading to beta-cell exhaustion, where persistent hyperglycaemia driving increased insulin secretion is unable to be matched by proinsulin biosynthesis in the beta-cell [31,32]. Degranulation can also be a result of increased ISGs degradation as in the case of Sorcs1 deficiency [33]. Many genes relevant to the ISG secretory pathway have recently been reviewed extensively by Liu and colleagues in the context of pathology [34]. These include the hydrolases that function both as an endopeptidase for prohormone maturation and as lysosomal proteases [35,36], vacuolar-type H^+^-transporting ATPases which regulate granule pH [37], and ZnT8 (SLC30A8), the key membrane transporter for zinc translocation into the maturing ISG [38]. Additionally, SNARE proteins and Rabs such as Vamp8 and Rab37 mediate ISG fusion at the site of insulin secretion [39]. Particularly interesting are the roles of cargo proteins within the ISG, which appear to have interdependent relationships. These include the well described soluble proteins carboxypeptidase E [40], VGF [41], the prohormone convertases PC1/3 and PC2, and the granin proteins chromogranin A [42], CgB and secretogranin II [43,44]. Figure 2 collates these ISG proteins and their localizations to immature and mature ISG. Mutations in these proteins can affect ISG formation, proinsulin processing, and glucose-stimulated insulin secretion, ultimately resulting in reduced ISG numbers and impaired secretion. However, loss of a single ISG cargo protein can drive compensatory behaviours in other ISG cargo proteins [42], suggesting ISG contents is a dynamic system.

## 2. Isolating the Insulin Granule

ISG isolation has long been used in the beta-cell physiology research, though the prioritisation of purity in the context of proteomic analyses is relatively new. Techniques used for the isolation of ISGs can essentially be separated into two categories, (a) differential subcellular fractionation using density gradients [45,46,47] and (b) immuno-based isolation [18,48,49,50]. In subcellular fractionation protocols, commercially available high-viscosity mediums such as Ficoll, Percoll, Optiprep and Nycodenz, or laboratory-prepared glycerol, sucrose or mannitol solutions are used to separate intact ISGs from cell lysates. These centrifugation techniques exploit physical properties including the size, density and/or shape of each subcellular compartment to separate ISGs from other organelles in collected fractions of various volumes. As these fractions are crude and undoubtedly contain contaminants, most studies employ the use of two or more subcellular fractionation steps to improve the purification of ISGs [45]. The main advantage of centrifugation techniques is that they are inexpensive and efficient [51], allowing researchers to obtain reasonably enriched ISG fractions within a few hours.

The second most common approach are immuno-based methods to enrich for ISGs [49,52], which exploit the tagging of proteins expressed in or on ISGs for isolation using immunoprecipitation. Often, this technique is used in conjunction with differential density gradients. For example, Hickey and colleagues employed the use of an Optiprep density gradient followed by Vamp2 immunoprecipitation to isolate ISGs from rat insulinoma INS-1 cells [48]. Immunoprecipitation offers the advantage of increased specificity to ISG compared to centrifugation techniques, however these methods are often more expensive and laborious, and rely on prior ISG protein knowledge. Interestingly, some proteins may be differentially expressed on ISGs. For example, CgB has heterogenous localization with insulin-positive granules in the INS-1 cell line [53]. Most importantly, immunoprecipitation of specific granule proteins that may be heterogeneously expressed would lead to the selective isolation of a specific ISG pool and the unknowing loss of information about the total ISG population.

On the other hand, immunoprecipitation could also selectively enrich for a non-ISG pool. In the same example, while Vamp2 immunoprecipitation may enrich for ISG, Vamp2 can also be expressed on Golgi recycling vesicles and endosomal membranes [54], and contamination of an ISG immunoprecipitation by these organelles cannot be disregarded. SG cargo proteins may also be present in pre-ISG compartments during the sorting process. Finally, immunoprecipitation methods also can be extended to protein pull-down studies which do not enrich ISG themselves, but instead immunoprecipitate interacting partners of known ISG proteins. Though these studies cannot offer a complete picture of the ISG proteome, they can offer an additional layer of insight into ISG protein functions and relationships [55,56,57].

**Figure 3:**
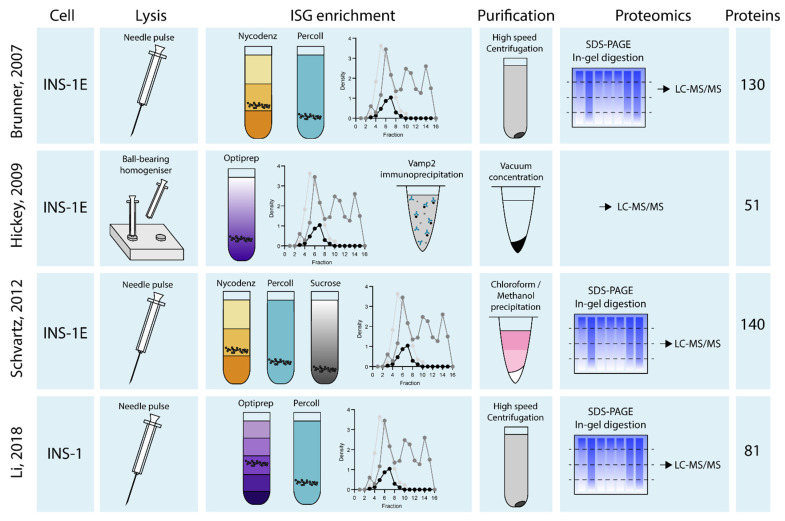
Schematic of insulin granule isolation and purification techniques across the 4 ISG proteomes. Rat insulinoma cells in all ISG proteomic studies are lysed before ISG enrichment through various density gradients or immunoprecipitations. ISG are then purified prior to proteomics analysis by LC-MS/MS to obtain list of ISG proteins. Data collated from Brunner et al., 2007, Hickey et al., 2009, Schvartz et al., 2012 and Li et al., 2018.

It is likely that some combination of both immuno-based and centrifugation methods will be necessary to obtain the purest ISG fractions. Techniques used by the studies that have attempted proteomic analysis of ISG isolations are summarised in Figure 3. There is currently no consensus on the optimal strategy for intact ISG isolation from whole beta-cells. Insulin SGs are intrinsically dynamic and distribute in many compartments of the beta-cell as they traffic through their maturation, secretion and degradation pathways [52]. Isolation of a pure ISG fraction is most challenging due to the association of ISG with proteins in multiple subcellular compartments [58,59], and previous proteomic analyses of ISGs notably include contaminating proteins from pre-granule compartments such as the ER and *trans*-Golgi network (TGN), as well as cytoskeletal and lysosomal proteins associated with the trafficking and degradation of ISGs respectively [58,59]. Mitochondrial contamination present in ISG purification methods is a major problem [60] and attempts to isolate ISGs often identify different mitochondrial proteins in insulin enriched fractions [48,50,59,61,62,63,64,65]. 

## 3. Identifying Insulin Granule Proteins

**Figure 4:**
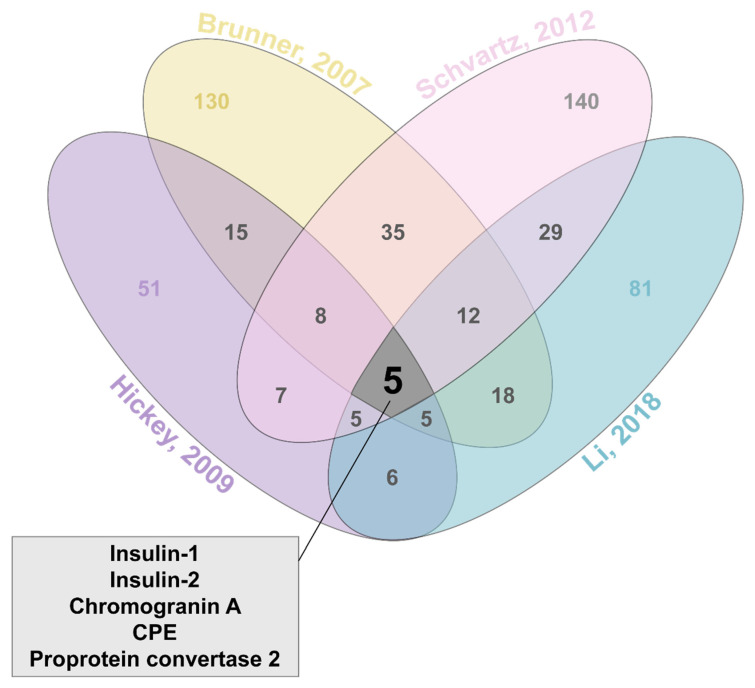
Venn diagram of overlapping proteins identified across the 4 ISG proteomes. Total of 5 proteins identified (box) in all proteomic analyses. Data collated from Brunner et al., 2007, Hickey et al., 2009, Schvartz et al., 2012 and Li et al., 2018.

Only four studies have attempted to investigate ISG proteins by proteomic analysis to date [48,59,62,65]. These studies employ various combinations of density gradient centrifugations, *in silico* analyses, and immunoprecipitation techniques (Figure 3). As a result, Li and colleagues identified 81 total ISG proteins from the INS-1 rat beta-cell line, while Schvartz et al. identified 140 ISG proteins, Hickey et al. identified 51 ISG proteins, and Brunner et al. identified 130 ISG proteins from the INS-1E rat beta-cell line (Figure 4). A complete list of overlap proteins can be found in Supplementary Table S1. Proteomic data obtained from these four studies on ISG proteins from INS-1 or INS1-E cells produced a total of 5 proteins that were consistently identified. These were: Insulin-1 (Ins1), Insulin-2 (Ins2), Carboxypeptidase E (CPE), Chromogranin-A (CgA) and Prohormone convertase 2 (PC2). Rat beta-cells synthesize two different forms of insulin encoded by the *Ins*1 and *Ins*2 gene that share 90% homology [66,67], hence two insulin forms found in these proteomes. Though different isolation techniques would influence the proteins identified, one would expect that using similar cell lines would result in more than a handful of proteins consistently identified across all four studies. 

Prior to ISG proteomics, Hutton et al. suggested ISGs may contain ~150 proteins using two-dimensional gel analysis of ISGs isolated from a rat islet tumour [46]. Approximately 30 specific proteins were described as ISG associated proteins before the first ISG proteome [62]. These proteins were individually classified primarily through cDNA screening and confocal microscopy. For example, the discovery of a well described ISG protein, the ZnT8 transporter was described as a pancreas-specific zinc transporter using RT-PCR on cDNA libraries with human tissue extracts [38]. Furthermore, ZnT8 was found to be localized specifically on ISGs through confocal microscopy of a fluorescent ZnT8 fusion protein expressed in INS-1 cells [38]. Similarly, phogrin was discovered as a membrane localized ISG protein through cDNA expression analysis and western blotting of phogrin with ISG enriched fractions [68]. These studies, among others were pivotal in uncovering different proteins that may modulate and affect insulin granule processes. Proteomics analysis of ISGs however provides an unbiased, comprehensive approach to the identification of multiple proteins simultaneously. Considering this however, all four studies lack the identification of these well-described ISG proteins such as ZnT8, any other zinc transporter and phogrin.

Here, we have classified the proteins identified in the ISG proteomes [48,59,62,65] into three groups: (i) intravesicular proteins, (ii) membrane proteins and (iii) other proteins: 

### 3.1. Intravesicular Proteins

The most consistently identified intravesicular proteins in the proteomic studies were the previously well-characterised ISG proteins insulin (Ins1 and Ins2), CPE, PC2 and CgA [9,40,42,69,70]. Discovery of proinsulin processing of labelled insulin [71] and CgA [72] have allowed subsequent studies to identify localization of PC1/3 [73], PC2 [74] and CPE [75] as ISG localized enzymes. While all proteomes identified PC2 and CPE, PC1/3 was discovered only in two studies [59,65]. Other intravesicular proteins identified were from the chromogranin-secretogranin protein family. CgA in particular was identified in all four studies, with full-length CgA believed to be important for the biogenesis of granules in beta-cells [76]. Interestingly, CgA knockout mice display a reduced islet number, beta-cell to alpha-cell ratio and plasma insulin levels [77]; however, they exhibit normal blood glucose levels, as a result of compensation from other granin proteins [42]. CgB has been suggested to not be specifically involved in granule formation but instead is essential in the secretion of insulin and other islet hormones such as somatostatin and glucagon [29]. However, through pulse-chase labelling of CgB, Bearrows et al. show that in the absence of CgB, there is a delay in proinsulin trafficking from the TGN followed by a reduction in nascent ISGs at the plasma membrane [44]. CgB was identified in three of the four ISG proteomes (all but Li et al.). Significantly, aside from the full-length granins, PC1/3 and PC2 also cleave granins to form active peptides [69,78]. Beta-granin is an example of a CgA derived peptide identified by Li et al. and is proposed to inhibit insulin secretion through unknown mechanisms [79]. This emphasises technical challenges in peptide identification in proteomics analysis, to differentiate the presence and eventual function of both granins and their derived peptides in future studies. 

Hydrolases were found in two of the proteomics analyses [48,62]. Cathepsins B and L were identified by Brunner et al. and are most intriguing as these proteins have been previously shown by electron microscopy to localise in immature ISGs, while cathepsin L alone remains in mature ISGs [36]. While some hydrolases have previously been described within ISGs [80,81], other hydrolases present in proteomic analysis may be appearing due to crinophagy processes of ISGs with lysosomes [62,82]. As such, further validation of hydrolase proteins will be essential to help elucidate their role in ISG biogenesis and processing. Particularly, the validation of cathepsins present in immature and mature ISGs demonstrates that these enzymes may follow sorting mechanisms out of immature ISGs via the mannose 6-phosphate receptor [36,83]. This adds weight to the ‘*sorting by retention*’ and ‘*sorting by exit*’ hypotheses in ISGs, in which immature ISGs may target proteins either for retention in maturing granules or exit towards the lysosome [36,62,84]. 

### 3.2. Membrane Proteins

A substantial proportion of ISG proteins identified by the proteomic analyses were membrane-bound or membrane-associated proteins. Of this group, the most commonly identified were synaptobrevin proteins (VAMPs), including Vamp3 [59,62,65], Vamp7 and Vamp8 [62]. VAMPs interact with their cognate t-SNAREs and other proteins that mediate the fusion of vesicles to the target membrane [85,86], which in turn interact with a variety of presynaptic proteins and q-SNAREs to form the complete SNARE complex [87,88,89]. Vamp2 was first described as an ISG localised v-SNARE protein [90] by cDNA cloning and confocal microscopy. Brunner et al. then identified Vamp2 in their proteomics analysis and following this, Hickey et al. used Vamp2 antibodies to immuno-purify ISGs. Surprisingly, Hickey et al. and Li et al. do not identify Vamp2 in their proteomes, with Hickey et al. suggesting that it and many other docking proteins potentially remained on the immunoaffinity beads [48]. If these membranal proteins were left unidentified, this may explain why fewer proteins (51) were identified in comparison to other proteomes.

Rab proteins were also found to be enriched with ISG fractions. Rab proteins are a family of GTPases from the Ras superfamily [91] that modulate several stages of vesicle trafficking and fusion of ISGs with the plasma membrane [92,93]. Through proteomic analysis and colocalisation imaging, Brunner’s study illustrated that both VAMP8 and Rab37 are novel ISG associated proteins that colocalise with ISGs of INS1-E cells [62]. Previous to this, only 30 proteins were described as ISG associated proteins in beta-cells [62] and information surrounding the trafficking of ISGs was limited. Their proteomic analyses and validation of novel proteins suggested a more complex trafficking process than previously established in beta-cells. Other SNARE complex proteins present in the proteomes include syntaxin5 and 12, (Stx5, Stx12) [59] and granuphilin [62]. However, these proteins are believed to be localised to the plasma membrane [94] and not on ISG membranes, suggesting that they were present in contaminant co-purification with ISG fractions. 

Many ATPase subunits were commonly identified in the four proteomic analyses, most notably the vacuolar-H^+^ ATPases (V-type). These V-type ATPases have been previously shown to be localized to ISGs in beta-cells [95], and are important in producing and maintaining a proton gradient by acidifying the granule [95,96,97]. This facilitates the maturation of ISGs [98] as well as maintaining a suitable pH for intravesicular enzymes [8,82]. Many other subunits of ATPases identified are lysosomal isoforms and should be validated as to whether they are genuine ISG proteins or proteins co-purified with ISGs.

### 3.3. Other Proteins

The remaining proteins identified with non-specific or unknown localization in ISGs are often grouped in these studies. These include cytoskeletal, cytoplasmic and organelle localized proteins. The cytoplasmic proteins identified range from mis-folding chaperones [48] and isomerases (PDIA3) [62] to N-ethylmaleimide sensitive fusion protein [59,65]. Whether these proteins are genuinely ISG-associated, or technical contaminants, requires further validation. Different cytoskeleton-associated proteins are found across all four proteomes. Alpha-centractin [65], alpha and beta-actin [48] and kinesin subunits [65] are some examples of cytoskeletal associated proteins identified. ISGs are transported along microtubules by kinesins [99] and cytoskeleton remodelling is critical for ISG trafficking during glucose-stimulated insulin secretion [100]. The presence of these proteins is therefore unsurprising, though are likely present due to co-purification of these proteins through the isolation of ISGs. Indeed, the presence of proteins localized to the ER, Golgi, mitochondria and lysosomes are also commonly observed across all four studies. Examples include Erp44 (ER), Glg1 (Golgi), SHMT (mitochondria) and Lamp1 (lysosomes) [59,65]. It is difficult to prevent the copurification of these proteins using present isolation techniques and their co-localisations with ISGs need further validation. 

The presence of isomerases and proteins involved in protein folding is quite surprising. Hickey et al. in particular find a striking number of chaperone proteins (~20% of proteins identified) [48]. Recent studies have shown that ER chaperone proteins are vital in proinsulin handling and insulin-like growth factor folding [101]; however, none of these ER-resident proteins have been shown to be localized in ISGs. Interestingly, Stanniocalcin-1 (STC1) or its precursors were found in three of the four proteomes (Li, Schvartz, Brunner). STC1 is found in many tissue types such as muscle, kidney, adrenal and lung [102]. Human STC1 protein is described as an uncoupler of oxidative phosphorylation in mitochondria [103], and has been implicated in apoptotic mechanisms and carcinogenesis [104]. Its function in beta-cells is not well understood, however; immunocytochemistry, and *in situ* ligand binding and hybridization [105] show that STC1 colocalizes with insulin in mouse pancreatic beta-cells. The abundance of these chaperones, alongside identification of proteins such as STC1, illustrates the importance of ISG proteomics as a rich source of data to potentially identify novel ISG proteins that may modulate different processes of ISG biogenesis, trafficking, and secretion. Altogether, these studies highlight the importance of developing improved purification techniques that restrict isolation of ISGs to granules post-sorting and packaging from the TGN, and before degradation.

## 4. Understanding ISG Function through the Proteome

Many aspects of ISG biosynthesis, processing, trafficking and secretion have been well reported [106], with the majority of studies focusing on individual protein effects on beta-cell function. Fewer studies use a broad view approach of ISG proteins, and their interactions and localisations. Efforts to identify exclusive ISG proteins in beta-cells remains scarce, and it is obvious that experimental methodology is the primary challenge. Proteomic analysis is appealing because it provides an unbiased approach to uncovering new ISGs proteins, and validation of targets will help understand mechanisms underlying beta-cell function. Indeed, the proteomics-based discovery of VAMP8 and Rab37 as ISG proteins by Brunner and colleagues resulted in the detection of a novel set of proteins that regulate fusion of ISGs to the plasma membrane, and thus established the paradigm for ISG exocytosis [62]. In a similar fashion, the identification of hydrolases [36] within the ISG lumen suggests there are still many facets of ISG recycling and degradation that remain unappreciated.

Intrinsic ISG behaviour is an intriguing concept, and the evidence for functionally distinct populations of mature ISG is growing. For a long time, ISG have been believed to exist in either a ‘readily releasable pool (RRP)’ or ‘reserve pool (RP)’ of granules within the beta-cell cytoplasm [107,108,109]. The presence of Rab37a effector protein granuphilin on ISG appears to regulate granule docking at the plasma membrane, interacting with Syntaxin-1A-Munc18-1 complexes [110], and contributing to the RRP. However, ISG docking has been found to be a limiting step in ISG exocytosis and is not a requirement for granule fusion, as it restricts ISG motility and is dysregulated in T2D [111]. In contrast, newcomer granules from the RP have been identified to exhibit high mobility [112] and fusion competence irrelative of docking [113]. Newcomer granules abundantly express Syntaxin-3, which interacts with Munc-13-1 and Vamp8 to mediate their priming and fusion states [114]. Newcomer ISG also appear to have high calcium sensitivity, fusing away from Syntaxin-1A and L-type Ca^2+^ channels [115]. Whether these distinct subpopulations can be distinguished by their proteome will be critical to understanding the physiological relevance for granule pools in ISG function. Indeed, there is some evidence for the existence of distinct mature granule subpopulations differentiated by the expression of surface markers synaptotagmins-7 and -9 [116].These ISG populations exhibit unique lipid compositions, calcium sensitivities, and even proprotein convertase protein distribution. Most significantly, relative proportions of these subpopulations are changed in diabetes, with the specific depletion of synaptotagmin-9 ISG observed in a model of T2D [116].

Recent studies have demonstrated that ISG age plays an important role in dictating secretion and degradation [16,17,18,117,118], with younger ISG preferentially secreted in first-phase glucose-stimulated insulin secretion. It is possible that changes in protein composition occur in aging ISG, controlling functional differences in these younger and older populations. Two unique strategies have since been used to identify age-distinct ISGs from beta-cells. The first is a fluorescent protein timer construct, syncollin-dsREDE5TIMER, that localises to the lumen of ISGs [18,119] and changes its emission spectra over time. Integrating this construct into beta-cells and then applying a technique termed fluorescence-assisted organelle sorting (FAOS), submicron vesicles are thus fluorescently-labelled for sorting [120]. In the second, Neukam et al. and Ivanova et al. employ the use of pulse-chase labelling of ISGs using either a SNAP or CLIP tag fused to insulin or phogrin respectively, followed by immuno-purification using fluorescent dye TMR [17,49]. The advantage of techniques that track syncollin or phogrin, as opposed to insulin, lie in the resulting exclusion of pre-sorting compartments within the beta-cell. Syncollin-dsREDE5TIMER is only red fluorescent in ISG from approximately 18 hours onwards, when ISG are distinctly mature, while phogrin-fused CLIP is immuno-precipitated by TMR only after its sorting in ISG. Neither technique has yet produced proteomic samples, potentially due to the challenge of separating old ISG from degradation pathways. Mature syncollin-dsREDE5TIMER is detectable within Lamp1-positive vesicles (data not shown), while Hoboth and colleagues also visualise SNAP-tagged ISG within multigranular autophagic bodies [16]. Neukam et al. attempt to mitigate this issue with the addition of a second immunoprecipitation step with Lamp2 and Syp1 to deplete apparent lysosomal contamination [49]. The optimisation of these methodologies will help expand our current understanding of the underpinnings regulating insulin secretion and beta-cell function.

## 5. Moving Forward

The four proteomic analyses examined in this review used combinations of density gradients to isolate ISGs. The use of additional ISG markers by Hickey et al. to further purify ISG is desirable in theory, but practically results in additional challenges. Of note, their use of Vamp2 immuno-isolation of ISG did not result in the identification of Vamp2 (or any other Vamp proteins) within their proteome. Indeed, with only five proteins identified across all four ISG proteomes, many established ISG-exclusive proteins such as PC1/3 [74], phogrin [68] and the ZnT8 transporter (SLC30A8, [38]) were not identified consistently or at all, confirming major technical limitations. There have also been enormous leaps in mass spectrometry technology since Brunner and colleagues first established an ISG proteome in 2007, and the exceedingly increased sensitivities from mass spectrometers and improved peptide search databases currently available will allow deeper proteome depth and accuracy [121,122]. Recent proteomics studies utilising library-based analyses techniques in mouse primary islets identified over 11,000 unique proteins using minimal starting material (unpublished data), suggesting similar database searches could generated and applied to the ISG proteomes to improve protein recognition. Li et al. additionally demonstrate the potential for novel protein discovery by utilising protein correlation profiling to match candidate proteins to known ISG markers based on Euclidean distance [59]. Recent development of different protein sequencing, such as nanopore technology [123] and fluorescent “protein fingerprinting” [124] may also facilitate new ISG protein identification.

The majority of ISG isolation studies focus solely on the mature ISG. Immature ISG isolation is considerably more difficult since immature ISGs lack the dense zinc core, and more closely associate with pre-sorting compartments. Though Chen et al. demonstrate the use of fixed Percoll percentages to enrich immature ISG [45] using density, both ER and TGN membrane proteins were found to be contaminating in those fractions. There is potential that the use of immature ISG-specific proteins could be further exploited to isolate immature ISGs. For example, PICK1 and ICA69 form a protein complex on immature granules, but only PICK1 persists in mature granules [125]. Similarly, clathrin is ‘*sorted-by-exit*’ from immature ISG, though is also present on non-ISG vesicles. Proteomic analyses of immature ISGs will improve our understanding of both sorting mechanisms at the TGN, and processing of the ISG itself during insulin maturation. Many beta-cell pathologies are intimately linked to ISG formation, despite the most common diabetes therapies targeting defective ISG secretion. Dysregulated ISG biogenesis leads to glucose intolerance *in vivo* [30,33,41,126], while increased proinsulin / insulin ratios are archetypical of diabetic patients and indicative of impaired processing within immature ISG [126,127].

Current ISG proteomes studies have only investigated rat insulinoma INS1 or INS1-E cell lines. This is most likely due to ease of culture and scaling to large starting material quantities, but it is important to consider how the ISG proteomes in mouse or human beta-cells may differ, potentially with the application of these methods to the MIN6 or EndoC-βH1 cell lines. Of the proteins with consensus across the ISG proteomes, all have human orthologs (INS, CHG, PCSK2, CPE), though humans only have a single insulin gene. Human beta-cell proteomics studies are also rare as they rely on precious and scarce material and are often subject to contamination by other endocrine cell types. Up to 707 potential beta-cell proteins have been identified [128,129,130], though it is yet unknown how many of those are ISG-specific.

Moreover, it will be critical to translate those techniques established within cell lines to primary cells to provide a more accurate snapshot of ISG proteins *in vivo*. There is potential to incorporate flow cytometry sorting techniques to isolate primary beta-cells, separated by an insulin-tagged fluorophore [131], zinc dyes or probes [132,133], or even NADPH autofluorescence [134,135] prior to ISG enrichment. Following this, the application of immunoprecipitation of select ISG markers [48], or dynamic fluorophores such as dsRedE5TIMER [18], will further allow ISG population separation. Once optimised, these methodologies would provide a standard for ISG proteomics that could be applied to multiple models of insulin-associated pathologies, including T2D.

A clean proteomic analysis of ISGs will provide a resource for more complete understanding of ISG sorting, processing, and trafficking. Currently, ISG proteomes are scarce, limited to rat insulinoma cell lines, and contain significant contamination. With the continued development of improved ISG isolation techniques, purification strategies and advancements in proteomics, ISG proteomes should be revisited, applied to different cell lines and ISG subpopulations to investigate and uncover novel players in the ISG secretory pathway.

## Data Availability

The figures and tables are original and not reproduced anywhere else.

## Supplementary Materials

The following are available online at https://www.mdpi.com/article/10.3390/metabo11050288/s1, Table S1: Overlap of proteins identified across the 4 ISG proteomes.
